# “COVID-19 as the equalizer”: Evolving discourses of COVID-19 and implications for medical education

**DOI:** 10.36834/cmej.70401

**Published:** 2020-09-23

**Authors:** Asia van Buuren, Vincent Tang, Maria Athina (Tina) Martimianakis

**Affiliations:** 1 Faculty of Medicine, University of Toronto, Ontario, Canada; 2 Department of Pediatrics, University of Toronto, Ontario, Canada; 3 Wilson Centre, University of Toronto, Ontario, Canada

## Introduction

The othering of patients is not a phenomenon unique to the COVID-19 pandemic. Indeed, this othering occurred during the HIV, SARS, and H1N1 outbreaks, impacting patient care and outbreak management.^1^^-^^4^ While the role of stigma during pandemics has already been explored and has highlighted societal inequities relevant to these conditions,^5^^,^^6^ stigma has not been a primary consideration during initial outbreak management, often causing relevant education to lag behind.^7^ As the early stages of the COVID-19 pandemic unfold, it provides us with a timely opportunity to begin to recognize and plan to address this gap.

During the early stages of the pandemic, initial discourses framed COVID-19 as an equalizer, placing emphasis on its universality.^8^^,^^9^ This framework justified government action and outbreak response organized along those lines. Authors and advocates have since criticized this discourse as it made invisible the experiences of marginalized populations.^10^^,^^11^ Indeed, approaches to COVID-19 through a lens of equality rather than through a lens of equity is, in itself, an injustice. Over time, as more people noticed how COVID-19 highlighted structural inequity, the societal discourse shifted. For example, the disproportionate impact of COVID-19 on Black populations in North America has underscored the structural inequities that these populations face.^12^^,^^13^ This shift in discourse from COVID-19 as an equalizer, to more recently, COVID-19 as exacerbating structural inequity, warrants exploration as it has potential to unearth unique vulnerabilities faced by populations in this pandemic. In this work, we will examine: 1) the discourse(s) that have emerged during the COVID-19 pandemic that highlight population-level inequities, and 2) the implications these discourses have for medical education, the learning environment, and the care patients receive from medical trainees.

## Methods

Critical discourse analysis (CDA) is a methodology that involves studying texts in order to understand how language constructs the possibility for making meaning and experiencing the world.^14^ In medical education, this methodology has been successful in identifying practices, linked to language use, that can advantage or disadvantage learners, faculty, and patients.^14^^,^^15^

Using a CDA approach,^14^ we will iteratively create an archive of public domain texts including newspapers, blog posts, government announcements, etc. that discuss COVID-19. While our data collection thus far has focused on implications for Canadian health care education and practice, the global nature of this illness warrants examination of texts circulating around the world.

The archive will be analysed for patterns of how COVID-19 has highlighted, reinforced, or dismantled structural inequity at the population level, emphasizing implications for medical education and learners. We will keep track of both positive and negative material effects of COVID-19 that are reported during the pandemic.^8^^,^^9^ After the discourse analysis has been completed, we will analyze our archive using an intersectional framework to better understand how language used during the pandemic may (re)produce othering. We will pursue an internal peer review process, seeking input from colleagues with expertise in CDA in order to continue to improve our work as it unfolds.

## Summary

Over the past few months, COVID-19 has highlighted challenges faced by marginalized populations across the globe. CDA serves as an effective medium to analyze the shifts in discourse that have since taken place. As the dialogue shifts from COVID-19’s universality to the recognition of the critical impact of structural inequities, a CDA analysis will allow an understanding of the implications for medical learners of normalizing and enhancing teaching surrounding these issues. We anticipate that this work will motivate further impetus for medical institutions to engage in teaching and advocacy around these issues, as the mandate of medical schools is to serve the populations that are closely tied to them. This study has the potential to impact medical education by improving how equity, diversity, and inclusion are taught to future generations of health care leaders.
